# Functional analyses of the C-terminal half of the *Saccharomyces cerevisiae* Rad52 protein

**DOI:** 10.1093/nar/gkt986

**Published:** 2013-10-25

**Authors:** Wataru Kagawa, Naoto Arai, Yuichi Ichikawa, Kengo Saito, Shusei Sugiyama, Mika Saotome, Takehiko Shibata, Hitoshi Kurumizaka

**Affiliations:** ^1^Department of Interdisciplinary Science and Engineering, Program in Chemistry and Life Science, School of Science and Engineering, Meisei University, 2-1-1 Hodokubo, Hino-shi, Tokyo 191-8506, Japan, ^2^Department of Applied Biological Science, Nihon University College of Bioresource Sciences, Fujisawa-shi, Kanagawa 252-0880, Japan, ^3^Laboratory of Structural Biology, Graduate School of Advanced Science and Engineering, Waseda University, 2-2 Wakamatsu-cho, Shinjuku-ku, Tokyo 162-8480, Japan and ^4^Cellular and Molecular Biology Laboratory, RIKEN, Wako-shi, Saitama 351-0198, Japan

## Abstract

The *Saccharomyces cerevisiae* Rad52 protein is essential for efficient homologous recombination (HR). An important role of Rad52 in HR is the loading of Rad51 onto replication protein A-coated single-stranded DNA (ssDNA), which is referred to as the recombination mediator activity. *In vitro*, Rad52 displays additional activities, including self-association, DNA binding and ssDNA annealing. Although Rad52 has been a subject of extensive genetic, biochemical and structural studies, the mechanisms by which these activities are coordinated in the various roles of Rad52 in HR remain largely unknown. In the present study, we found that an isolated C-terminal half of Rad52 disrupted the Rad51 oligomer and formed a heterodimeric complex with Rad51. The Rad52 fragment inhibited the binding of Rad51 to double-stranded DNA, but not to ssDNA. The phenylalanine-349 and tyrosine-409 residues present in the C-terminal half of Rad52 were critical for the interaction with Rad51, the disruption of Rad51 oligomers, the mediator activity of the full-length protein and for DNA repair *in vivo* in the presence of methyl methanesulfonate. Our studies suggested that phenylalanine-349 and tyrosine-409 are key residues in the C-terminal half of Rad52 and probably play an important role in the mediator activity.

## INTRODUCTION

Homologous recombination (HR) is an important mechanism for the accurate repair of DNA double-strand breaks. Defects in HR lead to mutations in the genome, as well as gross chromosomal rearrangements such as deletions, insertions and loss of heterozygosity, which could lead to genomic instability and cancer (1). HR consists of multiple reaction steps that are intricately coordinated by an array of proteins. Although many of the proteins have been well-characterized genetically, biochemically and structurally by themselves (2), the detailed molecular mechanisms by which these proteins interact with each other and promote HR remain obscure.

In yeast, Rad52 is an important factor in HR. Rad52 is a multifunctional protein involved in both the Rad51-dependent and Rad51-independent HR pathways. Increasing evidence suggests that the primary role of Rad52 in the Rad51-dependent HR pathways is to facilitate the loading of the Rad51 recombinase on replication protein A (RPA)-coated single-stranded DNA (ssDNA), which is referred to as the recombination mediator activity (3,4). Rad52 also efficiently promotes the annealing of complementary ssDNA (5). This activity may be important in the steps following strand invasion in the Rad51-dependent HR pathways, such as the second-end capture (6,7), and in the Rad51-independent HR pathways, including single-strand annealing (8).

Previous studies indicated that the N- and C-terminal halves of Rad52 have clearly distinct functions. The N-terminal half is highly conserved among Rad52 orthologs, and contains the self-association region. The isolated N-terminal half of the human Rad52 protein is capable of oligomerizing into a ring structure (9–12) and forms a DNA binding site that encircles the ring structure. Alanine scan mutagenesis has revealed the residues that are important for DNA binding (11,13,14). These structural and structure-based biochemical studies, along with single-molecule studies of Rad52 (15,16), have provided a framework for understanding the Rad52-mediated DNA annealing. In contrast, the C-terminal half of the human Rad52 protein has not been well characterized. The C-terminal regions of the Rad52 orthologs share poor sequence homology, and they are predicted to contain intrinsically disordered regions (17). In yeast and human Rad52, the RPA and Rad51 interacting regions have been mapped to the C-terminal halves (18–21). In yeast Rad52, a second DNA binding site has been found in the C-terminal third of the protein, which binds to both ssDNA and double-stranded DNA (dsDNA) (22). Truncation of the C-terminal region in yeast Rad52 impairs its ability to stimulate Rad51-mediated DNA strand exchange (20). Thus, the mediator function of yeast Rad52 is dependent on the C-terminal half. However, little information is available about the molecular mechanism underlying the mediator activity of Rad52.

To clarify the molecular roles of the C-terminal half of the *Saccharomyces cerevisiae* Rad52 protein, we prepared and biochemically characterized a C-terminal Rad52 fragment. We found that the Rad52 fragment disrupted Rad51 oligomers and hindered the formation of the Rad51–dsDNA complex. In addition, the alanine substitutions of phenylalanine-349 or tyrosine-409 abolished the observed biochemical activities of the Rad52 fragment. Both of these residues were critical for DNA repair *in vivo*, and for the mediator activity of Rad52 *in vitro*.

## MATERIALS AND METHODS

### Protein purification

The full-length *S. cerevisiae* Rad52, Rad51 and RPA proteins used in all *in vitro* experiments were expressed and purified as described previously (23,24). Rad52^233^^−504^, Rad52^233–^^504^ F349A and Rad52^233–^^504^ Y409A were expressed as hexahistidine-tagged proteins, using a pET15b derivative (pET15b-PS), in which the thrombin protease recognition site was replaced with a PreScission protease recognition site by site-directed mutagenesis. The protease recognition site was changed to minimize non-specific cleavage during hexahistidine tag removal. The *Rad52* genes were cloned between the *Nde*I and *Bam*HI sites of the pET15b-PS vector. These Rad52 derivatives were expressed in the *Escherichia coli* strain JM109(DE3), bearing a plasmid (pArg3Arg4, a gift from K. Sakamoto, RIKEN Systems and Structural Biology Center, Yokohama, Japan) to express low-abundance tRNAs. Each protein was purified from a 2-L LB culture that was initially incubated at 30°C. Protein expression was induced at an optical density (*A*_600_) of 0.7 with 0.5 mM 1-thio-β-d-galactopyranoside (final concentration), and the culture was incubated overnight at 18°C. The cells were harvested, resuspended in resuspension buffer (50 mM Tris-HCl, pH 7.8, 1.0 M KCl, 10% glycerol) containing 5 mM imidazole and lysed by sonication. The cell lysate was cleared of insoluble material by centrifugation at 30 190 x *g* for 30 min. The supernatant was mixed with 3 ml of nickel-nitrilotriacetic acid-agarose beads (Qiagen) and was incubated at 4°C with gentle mixing for 1 h. The mixture was poured into an Econo-Column, and the unbound cell lysate was drained out. Rad52 was eluted with 120 ml of resuspension buffer containing 50 mM imidazole. To remove the hexahistidine tag, the eluted protein was mixed with 0.5 U of PreScission protease per milligram of protein, and was immediately dialyzed against Rad52 purification buffer [20 mM Hepes-KOH, pH 7.5, 0.5 mM ethylenediaminetetraacetic acid (EDTA), 10% glycerol, 2 mM 2-mercaptoethanol] containing 0.15 M KCl. The dialyzed protein solution was collected, and the removal of the hexahistidine tag was confirmed by sodium dodecyl sulphate-polyacrylamide gel electrophoresis (SDS-PAGE). The protein solution was then passed through a 6-ml Q Sepharose column (GE Healthcare), and the flow-through fraction was diluted with water to adjust the KCl concentration to 0.1 M. The diluted fraction was directly applied to a 5-ml Affi-Gel Blue column (Bio-Rad). The column was washed with 100 ml of Rad52 purification buffer containing 0.1 M KCl, and the proteins were eluted with a 100-ml linear gradient of 0.1–1 M KCl in Rad52 purification buffer. The peak fractions were collected, dialyzed against Rad52 purification buffer containing 0.15 M KCl and concentrated to 0.5–2 mg/ml using a Vivaspin ultrafiltration device with a 10 kDa molecular weight cutoff (Sartorius). The concentrations of Rad52^233–^^504^ and its point mutants were determined by the absorbance at 280 nm, using an extinction coefficient of 4470 M^−^^1^ cm^−^^1^. The extinction coefficient was calculated with the ProtParam tool at the ExPASy Web site (http://web.expasy.org/protparam/).

### Size exclusion chromatography analysis

A 250-µl mixture containing 10 µM Rad51 and 20 µM Rad52^233^^−504^ was incubated at room temperature for 1 h. The 250-µl mixture was fractionated through a 24-ml Superdex 200 10/300 GL column (GE Healthcare) in SE Buffer, containing 20 mM Hepes-KOH, pH 7.5, 0.1 M KCl, 0.5 mM EDTA, 10% glycerol and 2 mM 2-mercaptoethanol. This analysis was performed identically for the Rad52^233–^^504^ F349A and Rad52^233–^^504^ Y409A mutants.

### Analytical ultracentrifugation

The Rad51–Rad52^233–^^504^ complex used for the sedimentation equilibrium analysis was prepared in a 250-µl mixture, containing 25 µM Rad51 and 50 µM Rad52^233–^^504^. The mixture was incubated at room temperature for 1 h. The complex was purified by chromatography on a 24-ml Superdex 200 10/300 GL column (GE Healthcare), using the SE Buffer described earlier. Peak fractions were collected and concentrated using an Amicon Ultra 10 ultrafiltration device (Millipore) until the absorbance at 280 nm reached 0.3. The complex was analyzed using a Beckman Optima XL-I instrument. The concentrated sample was spun in an 8-sector centerpiece, using a Beckman An-50Ti rotor. Equilibrium distributions were analyzed after 20 h of centrifugation, at 16 000 rpm and 20°C. For the molecular mass analysis, an estimated partial specific volume of 0.723 cm^3^/g and a solution density value of 1.03 g/cm^3^ were used. These values were calculated from the amino acid composition and the buffer components, respectively, using the SEDNTERP program (25). For the sedimentation equilibrium analysis of Rad52^233–^^504^, the equilibrium distribution was analyzed after 20 h of centrifugation, at 32 000 rpm and 20°C. The molecular mass of Rad52^233–^^504^ was calculated using the same partial specific volume and solution density values as those used for the complex.

### DNA binding by Rad51 in the presence of Rad52^233–^^504^

A 9-µl reaction mixture containing 2 µl of 10× reaction buffer (150 mM MOPS-KOH, pH 7.3, 100 mM magnesium acetate, 100 mM NaCl, 5 mM dithiothreitol), 1 µl of 20 mM CaCl_2_, 1 µl of 20 mM ATP, 1 µl of 10 µM Rad51 and 2 µl of Rad52^233–^^504^ (various concentrations) was incubated at 37°C for 15 min. To this mixture, 1 µl of ^32^P-labeled 60-mer ssDNA (5′ GGA ATT CGG TAT TCC CAG GCG GTC TCC CAT CCA AGT ACT AAC CGA GCC CTA TGC TGC TTG 3′) or dsDNA (same length and sequence) was added, and the solution was incubated for 15 min. The complexes were either fixed with 1 µl of 1% glutaraldehyde or treated with 2 µl of deproteinization solution (0.5% SDS, 10 mg/ml proteinase K) at 37°C for 15 min. The products were fractionated through a 1% Seakem GTG agarose (FMC BioProducts) gel in 0.5× Tris-borate-EDTA buffer for 2 h at 3.3 V/cm. The gels were dried, exposed to an imaging plate and visualized using an FLA7000 image analyzer (Fuji Film).

### Viability test after exposure to methyl methanesulfonate

Viability tests were performed using the yeast strain *S. cerevisiae* XS560-1C-1D2 (MATa, *rad52-8::TRP1, leu2-2, 112, trp1Δ, ura3-52, his3-Δ, can1*). The expression vector, pNS31, for the mutant and wild-type *RAD52* in *S. cerevisiae* contains the *ADH1* promoter and terminator, *ARSH4-CEN6* (a yeast centromere sequence, CEN, and an autonomous replication sequence, ARS) and the *LEU2* marker (26).

For the spot test, the synthetic dextrose (SD) liquid medium [2% glucose and 0.67% yeast nitrogen base without amino acids (Difco)] was supplemented with 2 µg/ml uracil and 2 µg/ml histidine. Cells from an overnight culture (10 ml) were concentrated to 1 ml by centrifugation at 200 × *g* for 3 min. The number of cells was counted under a microscope, using an improved Neubauer hemocytometer, and was adjusted to ∼10^5^ cells/µl. Aliquots (10 µl) of a 10-fold dilution series of each transformant were spotted onto SD plates containing 2 µg/ml uracil and 2 µg/ml histidine with 0.25, 0.59 or 1.18 mM methyl methanesulfonate (MMS). The plates were sealed with Parafilm and incubated at 30°C for 5 days.

For the quantitative test, the SD liquid medium was supplemented with 4 µg/ml adenine sulfate, 2 µg/ml uracil, 2 µg/ml histidine, 4 µg/ml lysine and 4 µg/ml tryptophan (and 6 µg/ml leucine for the YPH499 strain without the plasmid). Cells from an overnight culture (10 ml) were concentrated to 1 ml by centrifugation at 200 × *g* for 3 min. After the dilution of the cell cultures, the cells were spread onto freshly prepared SD plates containing the appropriate supplements, with MMS at 0, 0.13 (0.001%), 0.25 (0.002%), 0.59 (0.005%) or 1.18 mM (0.010%). The plates were sealed with Parafilm and incubated at 30°C for 7 days.

### DNA strand exchange assay

A ΦX DNA-based DNA strand exchange assay was performed, essentially as described (27). Briefly, a 10-µl reaction mixture, containing 35 mM MOPS-KOH, pH 7.1, 1 mM dithiothreitol, 50 µg/ml bovine serum albumin, 60 mM KCl, 2.5 mM ATP, 3 mM MgCl_2_, 9 µM Rad51, the indicated concentrations of Rad52, 2 µM RPA and 30 µM ΦX174 circular ssDNA, was prepared. All of the components (9 µl), except for the ssDNA, were mixed and incubated on ice for 45 min. The ssDNA (1 µl, 300 µM) was then added to the reaction mixture and incubated at 37°C for 10 min. Afterward, 1 µl of 300 µM dsDNA (ΦX174 RFI linearized by *Pst*I digestion) and 1 µl of 50 mM spermidine hydrochloride were added to the reaction mixture and incubated at 37°C for 90 min. The reaction was stopped by the addition of an equal volume of 1% SDS containing 1 mg/ml proteinase K. Deproteinization of the reaction mixtures was performed at 37°C for 20 min. After the addition of 0.2 volume of gel loading dye (0.2% bromophenol blue, 50% glycerol), the samples were fractionated in 1.0% agarose gels in Tris-acetate-EDTA buffer, stained with ethidium bromide for 60 min and then destained for at least 4 h in a large volume of H_2_O. Images were recorded with a Gel Doc XR system (Bio-Rad).

### ATPase assay

[α-^32^P]ATP (0.1 mM) was incubated with 1.0 µM RPA and 5.0 µM (in nucleotides) pUC119 circular ssDNA in reaction buffer (10 µl), containing 30 mM MOPS-KOH (pH 7.1), 7 mM MgCl_2_, 50 mM KCl, 1 mM dithiothreitol, 50 µg/ml bovine serum albumin and 0.02% (v/v) IGEPAL CA630 (SIGMA), at 37°C for 10 min. After the incubation, 1.0 µM Rad51 and the indicated concentrations of Rad52 were added to the reaction mixture, which was incubated further for 30 min. (The Rad51 and Rad52 were pre-incubated for 30–60 min on ice for complex formation, before they were added to the reaction mixture.) The reaction was terminated by adding 10 µl of stop solution (25 mM EDTA, 3 mM ATP, 3 mM adenosine diphosphate, 3 mM adenosine monophosphate). Aliquots (10 µl) of the sample were spotted on polyethyleneimine plastic film (POLYGRAM CEL 300 PEI for TLC, MACHERY-NAGEL GmbH & Co.) and developed in a mixture of 0.5 M lithium chloride and 1 M formic acid. The plastic film was exposed to a Phosphor Screen (GE Healthcare Bioscience) for 1.5 h, and then the radioactivity was quantitated by a Typhoon 9410 Variable Image Analyzer.

## RESULTS

### The C-terminal region of Rad52 disrupts oligomeric Rad51 and forms a stable complex with monomeric Rad51

**Figure 1.**
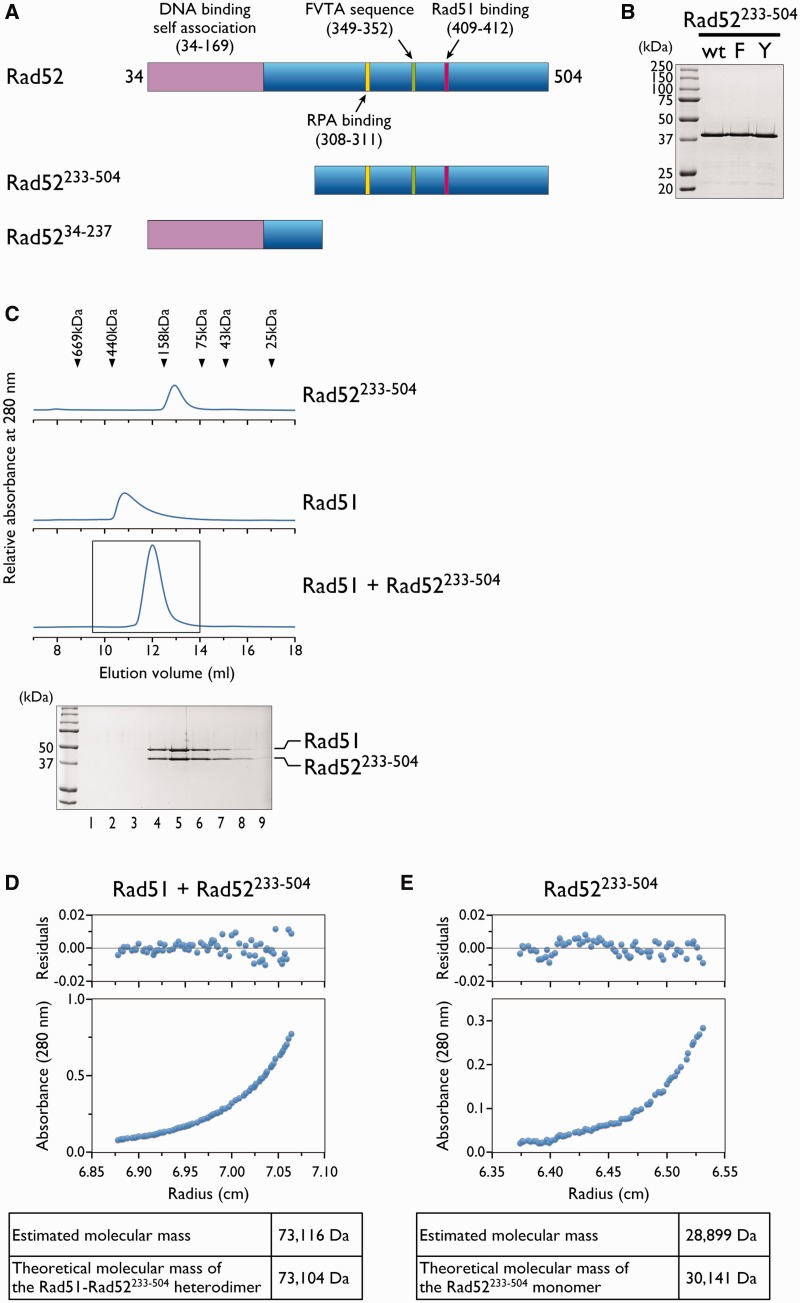
The C-terminal region of Rad52 depolymerizes Rad51. (**A**) Schematic representation of the Rad52 fragment. The functional regions in *S. cerevisiae* Rad52 are shown at the top. The region spanning amino acid residues 34–169 corresponds to the highly conserved region among Rad52 orthologs. Amino acid 34 is the N-terminus of the protein. (**B**) Purified Rad52^233–504^. The proteins (2 µg each) were fractionated through a 12% polyacrylamide gel. WT, F and Y denote wild-type, F349A and Y409A mutants, respectively. (**C**) Size exclusion chromatography analyses of Rad52^233–504^, Rad51 and a mixture of the two proteins. The elution profiles of the proteins from a Superdex 200 gel filtration column were monitored by the absorbance at 280 nm. The boxed portion (9.5–14 ml) containing the peak was fractionated in 0.5 ml portions, and each fraction was analyzed by SDS-PAGE (lanes 1–9), as shown. Sedimentation equilibrium analysis of the Rad51–Rad52^233–504^ complex (**D**) or Rad52^233–504^ (**E**). The distribution of the complex in the cell reached equilibrium after 20 h of centrifugation at 16 000 rpm (Rad51–Rad52^233–504^ complex) or 32 000 rpm (Rad52^233–504^). The distribution of the complex was determined by UV scanning of the cell (280 nm) at incremental steps, as plotted in the lower panel. The radius indicates the distance from the center of the rotor. The upper panel shows the residual differences between the experimental data and the fit for each data point. The estimated molecular mass in the table below is the result of the molecular mass analysis, in which the data were fitted to an ideal single-component model. The theoretical molecular mass was calculated from the amino acid sequences of Rad52^233–504^ and Rad51.

The precise stoichiometry of the Rad52–Rad51 interaction is presently unknown. Because Rad51 and Rad52 both form oligomeric complexes with various numbers of monomers (28,29), it is difficult to experimentally determine the precise stoichiometry of Rad52 and Rad51 in the complex, using full-length proteins. To circumvent this problem, we prepared a Rad52 fragment (Rad52^233–^^504^) containing only the C-terminal half of the protein, which includes the previously identified Rad51 binding region, but lacks the N-terminal region required for self-association (Figure 1A and B). Size exclusion chromatography of the purified Rad52^233^^−504^ (Figure 1C) revealed that the protein eluted between the 75 and 158 kDa molecular weight markers. Rad52^233–^^504^ was much smaller than the full-length protein, which eluted at the void volume (data not shown). When Rad52^233–^^504^ was mixed with Rad51 and analyzed by size exclusion chromatography, we observed a single peak that clearly indicated a molecular mass larger than that of Rad52^233–^^504^, but significantly smaller than that of Rad51 (Figure 1C). The fact that Rad51 alone forms oligomeric complexes (Figure 1C) indicates that Rad52^233–^^504^ is capable of disrupting oligomeric Rad51. An SDS-PAGE analysis of the peak fractions revealed the co-elution of Rad52^233–^^504^ and Rad51 (Figure 1C). The band intensities of both proteins were similar, suggesting that Rad52^233–^^504^ and Rad51 formed a complex with 1:1 stoichiometry. To determine the precise number of Rad52^233–^^504^ and Rad51 molecules in the complex, the peak fractions were concentrated and analyzed by analytical ultracentrifugation. The sedimentation equilibrium analysis revealed that Rad52^233–^^504^ and Rad51 formed a complex with a molecular mass of ∼73 kDa (Figure 1D), which is close to the size of a heterodimer. Rad52^233–^^504^ alone had a molecular mass of ∼30 kDa, indicating that it was a monomer (Figure 1E). These results demonstrated that the C-terminal region of yeast Rad52 is capable of disrupting Rad51 oligomers and forming a heterodimeric complex with the Rad51 monomer.

Notably, the molecular mass estimates of Rad52^233–^^504^ and the Rad51–Rad52^233–^^504^ complex from the size exclusion chromatography were significantly larger than those from the analytical ultracentrifugation analysis. Considering that the shape of the molecule can affect its behavior in size exclusion chromatography, the higher than expected molecular mass of Rad52^233–^^504^ and the Rad51–Rad52^233–^^504^ complex might indicate an elongated shape of Rad52^233–^^504^. This interpretation appears reasonable, given that the C-terminal half of Rad52 is predicted to contain intrinsically disordered regions, which generally have more elongated shapes than folded globular regions.

### The C-terminal region of Rad52 hinders the formation of the Rad51–dsDNA complex

**Figure 2.**
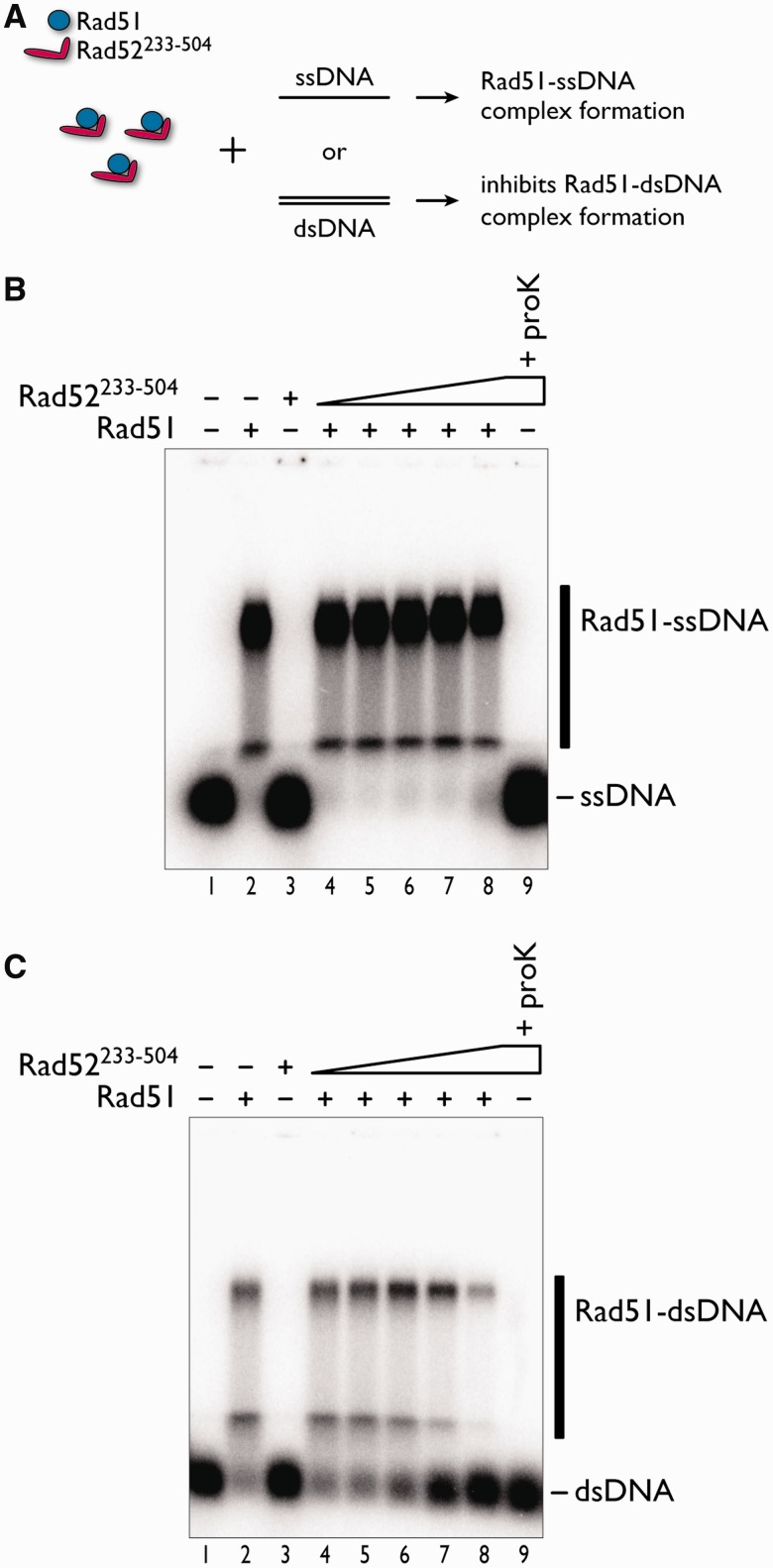
Effects of Rad52^233–504^ on the formation of Rad51–ssDNA and Rad51–dsDNA complexes. (**A**) Schematic representation of the assay. ssDNA (1 µM in nucleotides) (**B**) or dsDNA (1 µM in nucleotides) (**C**) was added to the reaction mixture containing Rad52^233–504^ and Rad51 (1 µM). Products were stabilized by glutaraldehyde fixation and fractionated through an agarose gel. The fixed Rad51–DNA complexes migrated to two major locations in the gel (B and C, lane 2). Increases in the Rad52^233–504^ concentration caused greater increases in the unbound dsDNA, rather than the unbound ssDNA (B and C, compare lanes 6–8). The Rad52^233–504^ concentrations were 0.5 µM (lane 4), 1 µM (lane 5), 2 µM (lane 6), 4 µM (lane 7) and 8 µM (lanes 3, 8 and 9). Lanes 3 and 9 are identical, except for the addition of proteinase K after glutaraldehyde fixation.

To further characterize the function of the C-terminal half of yeast Rad52, we examined the effect of Rad52^233–^^504^ on the assembly of the Rad51 oligomer on ssDNA or dsDNA. The exclusion of the N-terminal half of Rad52 circumvents the complication of observing a DNA binding competition between Rad51 and the N-terminal DNA binding domain of Rad52. In the assay, Rad51 and Rad52^233–^^504^ were pre-incubated together, and then ssDNA or dsDNA was added to the reaction mixture containing the two proteins (Figure 2A). Before electrophoretic separation of the products, the reaction mixtures were treated with a protein-DNA crosslinking reagent (glutaraldehyde), which was required to stabilize the Rad51–DNA complexes (Supplementary Figure S1). We found that Rad52^233–^^504^ did not appreciably inhibit the formation of the Rad51–ssDNA complex, as judged from the similar amounts of the complex and the absence of the unbound ssDNA (Figure 2B, lanes 4–8). In contrast, increasing concentrations of Rad52^233–^^504^ clearly inhibited the formation of the Rad51–dsDNA complex, as shown by the decreasing amounts of the Rad51–dsDNA complex and the increasing amounts of the unbound dsDNA (Figure 2C, lanes 6–8). These results suggested that the C-terminal half of Rad52 hinders Rad51 oligomer assembly on dsDNA. Importantly, Rad52^233–^^504^ did not exhibit DNA binding activity (Figure 2B and C, lane 3) in this assay, which seemingly contradicts the previous finding that the C-terminal half of Rad52 contains a DNA binding site (22). We found that in a buffer without Mg^2+^ and Ca^2+^ ions, Rad52^233–^^504^ bound to both ssDNA and dsDNA (Supplementary Figure S2), suggesting that divalent ions may inhibit the DNA binding activity of Rad52^233–^^504^.

We next examined the effect of adding Rad52^233–^^504^ to pre-formed Rad51–ssDNA and Rad51–dsDNA complexes. In this assay, Rad51 was pre-incubated with ssDNA or dsDNA, and then Rad52^233–^^504^ was added to the reaction mixture. The amounts of the Rad51–DNA complexes did not change with increasing concentrations of Rad52^233–^^504^, suggesting that Rad52^233–^^504^ did not destabilize either of the complexes (Supplementary Figure S3).

### Phenylalanine-349 and tyrosine-409 of Rad52 are essential for the disruption of the oligomeric Rad51

**Figure 3.**
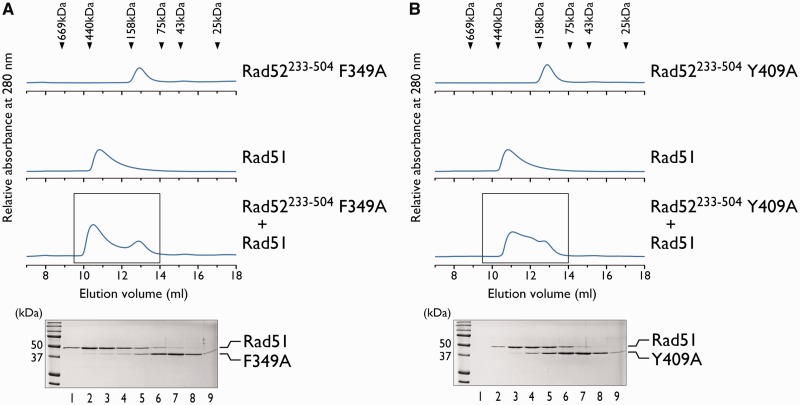
F349 and Y409 of Rad52 are essential for the interaction with Rad51. Size exclusion chromatography analyses of Rad52^233–504^ F349A-Rad51 (**A**) and Rad52^233–504^ Y409A-Rad51 (**B**) mixtures. The elution profiles of the proteins from a Superdex 200 gel filtration column were monitored by the absorbance at 280 nm. The boxed portion (9.5–14 ml) containing the peak was fractionated in 0.5 ml portions, and each fraction was analyzed by SDS-PAGE (lanes 1–9), as shown below.

To examine the Rad52–Rad51 interaction in more detail, we investigated whether the Rad51 polymerization motif, within the C-terminal half of Rad52, plays a role in the interaction. The amino acid sequence of the yeast Rad51 polymerization motif is FVTA (Rad51^144–^^147^). The crystal structure of the yeast Rad51 filament revealed that the phenylalanine residue interacts with the neighboring Rad51 monomer in a manner resembling a ‘ball and socket’ joint (30,31). In Rad52, the FVTA sequence (Rad52^349–^^352^) resides between the previously identified RPA and Rad51-interacting regions (Figure 1A). To determine whether the corresponding phenylalanine residue (F349) of Rad52 is involved in the interaction with Rad51, we examined the interaction between Rad51 and a Rad52^233–^^504^ mutant containing the phenylalanine to alanine substitution (Rad52^233–^^504^ F349A), by size exclusion chromatography, as described earlier. Unlike the Rad52^233–^^504^ without the point mutation, the Rad52^233–^^504^ F349A mutant did not co-elute with Rad51 (Figure 3A), indicating that they did not stably interact with each other. This result suggested that the phenylalanine residue of Rad52 plays an important role in the stable association with Rad51.

The FVTA sequence of Rad52 is ∼60 amino acid residues apart from the previously identified Rad51 binding region (20). To determine whether this region is also involved in the disruption of the Rad51 oligomer, we prepared a Rad52^233–^^504^ mutant containing the tyrosine to alanine substitution (Rad52^233–^^504^ Y409A) and examined its interaction with Rad51 by size exclusion chromatography. Similar to Rad52^233–^^504^ F349A, Rad52^233–^^504^ Y409A did not co-elute with Rad51 (Figure 3B), indicating that the tyrosine residue is also important for the stable physical interaction with Rad51. The F349A and Y409A mutations did not significantly disrupt the structure of Rad52^233–^^504^, as judged from the CD spectra (Supplementary Figure S4), suggesting that the loss of Rad51 binding was not the result of a disruption of the binding interface. Thus, our results demonstrated that Rad52 has at least two important interaction sites for Rad51 binding.

### Yeast cells bearing the Rad52 F349A and Y409A mutations are defective in DNA repair

**Figure 4.**
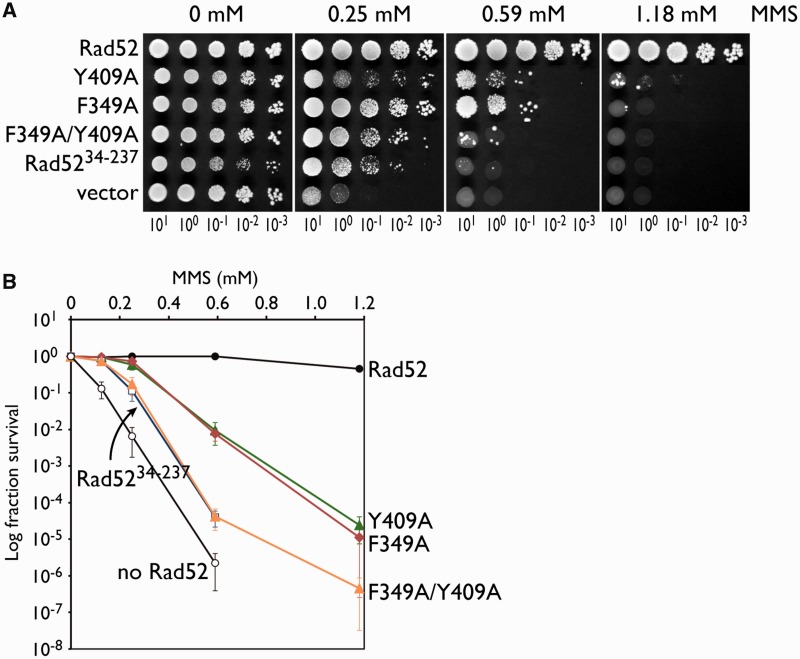
Mutations in F349 and Y409 of Rad52 impair its ability to repair MMS-induced DNA damage. Wild-type and mutant Rad52 proteins were expressed from a single-copy plasmid in *rad52Δ* haploid transformants, and their abilities to complement the MMS sensitivity exhibited by the haploid strain were examined by a spot assay. (**A**) Tenfold serial dilutions of the transformants were spotted onto plates containing 0, 0.25, 0.59 or 1.18 mM MMS and incubated at 30°C for 5 days. (**B**) Quantitative representation of the MMS sensitivities of the haploid strains. The double mutant displayed higher sensitivity toward MMS, as compared with the single mutants, and was similar to a Rad52 deletion mutant lacking the entire C-terminal half (Rad52^34–237^). Black circle, *RAD52*; green triangle, *rad52 Y409A*; black diamond, *rad52 F349A*; orange triangle, *rad52 F349A/Y409A*; white square, *rad52^34–237^*; and white circle, vector.

To establish the importance of the Rad52 F349 residue *in vivo*, we replaced the chromosomal *RAD52* gene with the *rad52 F349A* allele, and tested the mutant strain for its MMS sensitivity by a spot assay and a quantitative assay. Increasing the MMS concentration resulted in a significant decrease in the survival of the *rad52 F349A* mutant (Figure 4A and B). The MMS sensitivity exhibited by the *rad52 F349A* mutant was similar to that of the *rad52 Y409A* mutant. When a strain bearing both point mutations (the *rad52 F349A/Y409A* double mutant) was tested, we observed a higher level of MMS sensitivity. These observations suggested that F349 and Y409 are both important for the function of Rad52 in DNA repair *in vivo*. Interestingly, the MMS sensitivity of the *rad52 F349A/Y409A* double mutant was similar to that of a strain carrying a C-terminally truncated *RAD52* gene (*rad52^34^*^−^*^237^*, Figure 1A). This result suggested that F349 and Y409 are responsible for the core functions of the C-terminal half of Rad52.

### The mediator function of Rad52 requires F349 and Y409

**Figure 5.**
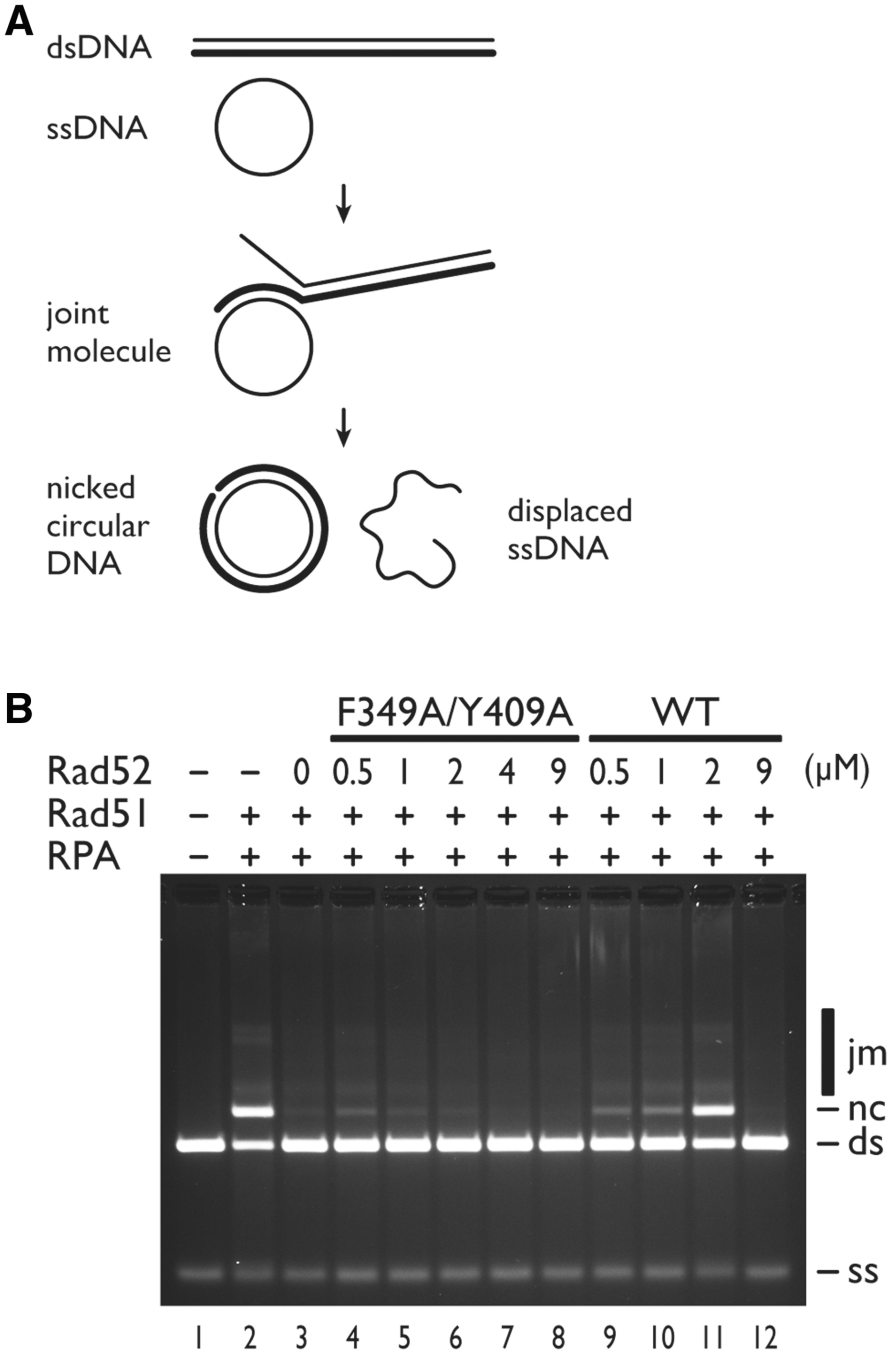
Recombination mediator activity of the Rad52 F349A/Y409A mutant assessed by a DNA strand exchange assay. (**A**) Schematic representation of the DNA strand exchange reaction. Circular ssDNA (ss) and linear dsDNA (ds) base pair to form a joint molecule (jm), in a process called homologous pairing. The joint molecule is converted into a nicked circular DNA (nc) by DNA strand exchange. (**B**) A standard reaction was performed by pre-incubating Rad51 (9 µM before the addition of dsDNA) with ssDNA (30 µM before the addition of dsDNA), followed by the addition of RPA (2 µM before the addition of dsDNA) and then by the addition of 1 µl dsDNA (30 µM final concentration) to start the reaction (lane 2). Co-incubation of Rad51 and RPA resulted in the severe inhibition of the DNA strand exchange reaction (lane 3). The inclusion of the indicated amounts of Rad52 (lanes 9–12) or the Rad52 F349A/Y409A mutant (lanes 4–8) led to various degrees of reaction restoration. The indicated concentrations of Rad52 are those before the addition of dsDNA.

To biochemically demonstrate that F349 and Y409 play important roles in the mediator activity of Rad52, we used the plasmid DNA-based DNA strand exchange assay established by Sung’s laboratory (27). In this assay, the ability of Rad51 to promote the formation of heteroduplexes (joint molecule and nicked circular DNA) between the circular ssDNA and linear dsDNA in the presence of RPA and Rad52 was monitored (Figure 5A). The wild-type Rad52 protein stimulated product formation most effectively at a Rad52-Rad51 molar ratio near 1:5 (∼2 µM Rad52, 10 µM Rad51) (Figure 5B, compare lane 3 with lanes 9–11), but at a molar ratio close to 1:1 (Figure 5B, lane 12), no stimulation was observed. These results are similar to those previously reported (27), and indicate that Rad52 overcomes the inhibitory effect of RPA on the formation of the Rad51 presynaptic filament. When Rad52 was replaced with the full-length F349A/Y409A double mutant, the mutant was clearly defective in stimulating the heteroduplex formation (Figure 5B, compare lanes 4–8 with lanes 9–11).

**Figure 6.**
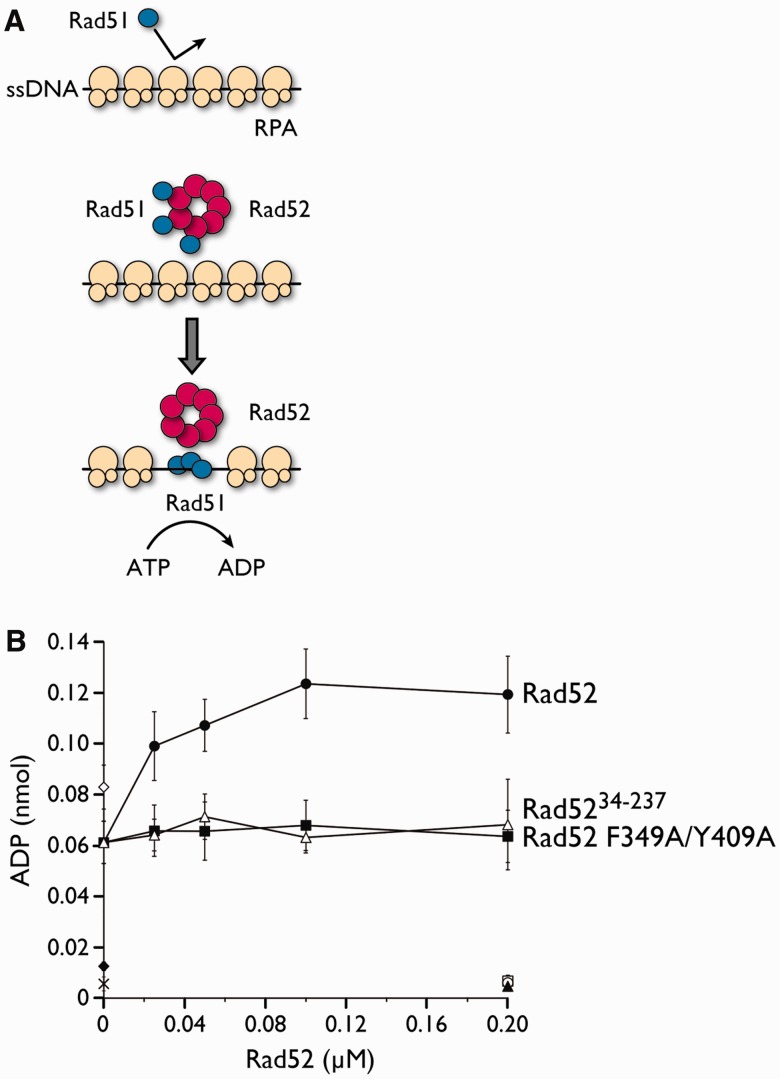
Recombination mediator activity of the Rad52 F349A/Y409A mutant assessed by an ATPase assay. (**A**) Schematic representation of the ATPase assay. The mediator activity of Rad52 was observed by monitoring the ATPase activity of Rad51 in the presence of RPA-bound ssDNA. The Rad52-mediated replacement of RPA with Rad51 on ssDNA would result in increased levels of ATP hydrolysis by Rad51. In the reaction, RPA was preincubated with circular ssDNA, followed by the addition of Rad52 and Rad51, which were preincubated together before addition. (**B**) Graphical representation of the ATPase assay. The amount of ADP produced as a function of Rad52 concentration was plotted. Black circle, Rad52; black square, Rad52 F349A/Y409A; white triangle, Rad52^34–237^; white diamond, Rad51 alone (no RPA); black diamond, RPA only; cross mark, no protein; white circle, Rad52 alone; white square, Rad52 F349A/Y409A alone; and black triangle, Rad52^34–237^ alone.

We also used our previously established ATPase assay to monitor the mediator activity of Rad52 (26). The assay measures the ATPase activity of Rad51 in the presence of RPA-coated ssDNA and Rad52. Efficient ATP hydrolysis is dependent on the displacement of RPA and the loading of Rad51 onto ssDNA, which are both promoted by Rad52 (Figure 6A). Using this assay, we examined whether the F349A/Y409A double mutation affects the mediator activity of Rad52. In the presence of the wild-type Rad52 protein, the ATP hydrolysis by Rad51 was stimulated ∼2-fold, as compared with the reaction without Rad52. The hydrolysis reached a plateau between 0.1 and 0.2 µM Rad52, which corresponds to a Rad52-Rad51 molar ratio between 10:1 and 5:1. In contrast, the Rad52 F349A/Y409A mutant and the C-terminally truncated Rad52 mutant (Rad52^34–^^237^) were completely defective in stimulating the ATPase activity of Rad51 (Figure 6B). The results from both assays revealed that the phenylalanine and tyrosine residues of Rad52 play important roles in assembling Rad51 onto ssDNA.

## DISCUSSION

**Figure 7.**
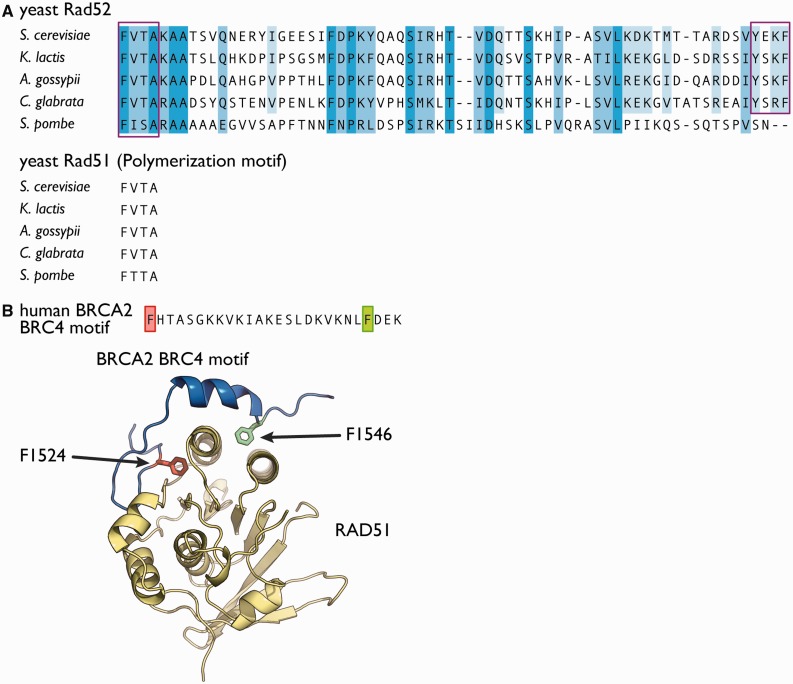
Conservation of F349 and Y409 in yeast Rad52 orthologs. (**A**) The C-terminal region of *S. cerevisiae* Rad52, spanning amino acid residues 349–412, was aligned with the homologous regions of the yeast Rad52 orthologs from *Kluyveromyces lactis*, *Ashbya gossypii*, *Candida glabrata* and *Schizosaccharomyces pombe*. The boxed regions indicate the FVTA sequence and the previously identified Rad51-binding region (20). The polymerization motifs of Rad51 from the corresponding yeast species are shown below. (**B**) Crystal structure of the fourth BRC repeat (BRC4) from the human BRCA2 protein complexed with Rad51 (PDB ID, 1N0W). BRC4 (blue) forms two important interactions with Rad51 (yellow). Both interactions involve phenylalanine residues (F1524 and F1546) that are ∼20 amino acid residues apart. F1524 is part of the FHTA motif that is highly similar to the FVTA sequences used by yeast Rad51 and Rad52.

In the presence of yeast Rad52 fragment that lacks the N-terminal self-association and DNA binding domains, we observed the depolymerization of Rad51, and the stoichiometric association between the Rad52 fragment and Rad51. The phenylalanine-349 and tyrosine-409 residues of Rad52 were critical for the interaction with Rad51. The phenylalanine residue is highly conserved among the yeast orthologs of Rad52 (Figure 7A) and resides within a four amino acid sequence (FVTA) that is also present in Rad51. The FVTA sequence is highly conserved among yeast Rad51 orthologs (Figure 7A) and is used for homo-oligomerization. The fact that phenylalanine-349 is important for the depolymerization of Rad51 suggested that the FVTA sequence in Rad52 may mimic the polymerization motif of Rad51 to antagonize the Rad51 oligomerization. A similar motif is also used by the breast cancer type 2 susceptibility protein (BRCA2), which functions as a recombination mediator in higher eukaryotes. The crystal structure of the fourth BRC repeat of human BRCA2 complexed with the human Rad51 protein revealed that the phenylalanine residue in the motif is a key residue for the interaction with Rad51 (32). The residue fits into the hydrophobic pocket of Rad51, which serves as an interface for the oligomerization between individual Rad51 monomers (Figure 7B). A similar interaction may occur between the phenylalanine-349 residue of Rad52 and Rad51.

The depolymerization of Rad51 by the C-terminal region of Rad52 is consistent with the mediator activity of Rad52. Because Rad52^233–^^504^ did not inhibit the formation of the Rad51–ssDNA complex (Figure 2), one possible mechanism is that the C-terminal region of Rad52 loads Rad51 onto ssDNA as the monomer. Furthermore, the ability of Rad52 to inhibit Rad51 loading onto dsDNA may also play an essential role in the mediator activity of Rad52. To gain better understanding of the underlying mechanisms by which yeast Rad52 facilitates the loading of Rad51, it will be important to clarify how the multiple C-terminal regions of Rad52 proteins function together in the assembly of the Rad51 oligomer on ssDNA. Although the oligomerization state of Rad52 during its mediator activity is not known, it probably functions as an oligomeric complex. Consistently, a previous study found that a Rad52 fragment lacking the conserved N-terminal region was inefficient in stimulating the Rad51-mediated DNA strand exchange (22). It is interesting to note that many BRCA2 orthologs also have multiple Rad51 binding sites (BRC repeats), with slight variations in the amino acid sequences and different affinities for Rad51 (33–38). The means by which these binding sites cooperate together in the mediator activity of BRCA2 are also a subject that awaits further investigation. Future studies of yeast Rad52 and mammalian BRCA2 may reveal the common mechanistic features of these recombination mediators.

Variations of the FVTA sequence are observed in several HR proteins that regulate the function of Rad51, and it appears to be a general interaction module in HR. RECQL5, a mammalian DNA helicase that directly interacts with Rad51 and suppresses its DNA pairing activity, reportedly possesses a BRCA2 BRC repeat-like region (39). Mutations in the corresponding four amino acid sequence of RECQL5 resulted in compromised functions, both *in vivo* and *in vitro*. A variant form of the BRC repeat is also observed in the yeast Srs2 and Sgs1 helicases, which have anti-recombinase activities similar to that of RECQL5 (39). These observations highlight the general use of the motif in the negative and positive regulators of Rad51.

Finally, the function of the C-terminal half of Rad52 may differ among eukaryotic species. Ascomycete fungi, including *S. cerevisiae*, lack BRCA2 and apparently rely on Rad52 for the recombination mediator function. The FVTA motif in *S. cerevisiae* Rad52, which was demonstrated to be important for the mediator activity of the protein, is conserved in the close relatives of *S. cerevisiae* (Figure 7A). Interestingly, the Rad52 proteins from non-yeast ascomycete fungi, such as *Neurospora*, *Coccidioides* and *Histoplasma*, lack the FVTA motif, despite their expected roles as mediators. These observations suggest that the amino acid sequences or motifs of Rad52 involved in the mediator activity may be variable. Therefore, in eukaryotic species containing BRCA2, it is unclear whether Rad52 functions as a recombination mediator. To our knowledge, the mammalian Rad52 proteins lack the FVTA motif (or a similar motif). *In vitro* studies have failed to detect the mediator activity of human Rad52 (Kagawa W., unpublished observations). Thus, in these species, Rad52 may not function as a recombination mediator, and BRCA2 fulfills the role instead. Another possibility is that the Rad52 in these species uses a different recombination mediator mechanism, which may involve post-translational modifications or an interaction with another protein (4). Intriguingly, human Rad52 contains a Rad51 binding site in its C-terminal half (21), but its biological significance remains obscure. Thus, more detailed studies of the C-terminal half of Rad52 from eukaryotic species containing BRCA2 are required to understand the molecular mechanisms underlying the function of Rad52 in HR.

## SUPPLEMENTARY DATA

Supplementary Data are available at NAR Online.

## FUNDING

The Grants-in-Aid from the Japanese Society for the Promotion of Science (JSPS) (in part) and the Ministry of Education, Culture, Sports, Science and Technology (MEXT), Japan, (to H.K., T.S.); and the Waseda Research Institute for Science and Engineering supported (to W.K. and H.K.). Funding for open access charge: Waseda University.

*Conflict of interest statement*. None declared.

## Supplementary Material

Supplementary Data
