# Supplementary material for: Functional analyses of the C-terminal half of the Saccharomyces cerevisiae Rad52 protein
Source: Nucleic Acids Res. 2013 Oct 25;42(2):941–51. doi: 10.1093/nar/gkt986 (PMC3902949; doi:10.1093/nar/gkt986)
Supplement: Supplementary Data [file supp_42_2_941__index.html]

Functional analyses of the C-terminal half of the Saccharomyces cerevisiae Rad52 protein — Functional analyses of the C-terminal half of the Saccharomyces cerevisiae Rad52 protein — Supplementary Data 

# Functional analyses of the C-terminal half of the *Saccharomyces cerevisiae* Rad52 protein

## Supplementary Data

files

**Files in this Data Supplement:**

- Supplementary Data - pdf file
