# Supplementary material for: Functional analyses of the C-terminal half of the Saccharomyces cerevisiae Rad52 protein
Source: Nucleic Acids Res. 2013 Oct 25;42(2):941–51. doi: 10.1093/nar/gkt986 (PMC3902949; doi:10.1093/nar/gkt986)
Supplement: Supplementary Data [file supp_gkt986_nar-01440-v-2013-File009.pdf]

Functional analyses of the C terminal half of the Saccharomyces cerevisiae Rad52 protein

Supplementary Information

Wataru Kagawa, Naoto Arai, Yuichi Ichikawa, Kengo Saito, Shusei Sugiyama, Mika Saotome, Takehiko Shibata and Hitoshi Kurumizaka

SUPPLEMENTARY METHODS

Rad51 DNA binding assay

The reaction mixture was essentially identical to that used in the Rad51 DNA binding assay in the presence of Rad52$^{233\text{-}504}$. A 9-μl reaction mixture, containing 2 μl of 10x reaction buffer (150 mM Mops-KOH, pH 7.3, 100 mM magnesium acetate, 100 mM NaCl, 5 mM DTT), 1 μl of 20 mM $CaCl_2$, 1 μl of 20 mM ATP, and 1 μl of 10 μM ^{32}P-labeled 60-mer ssDNA (5' GGA ATT CGG TAT TCC CAG GCG GTC TCC CAT CCA AGT ACT AAC CGA GCC CTA TGC TGC TTG 3') or dsDNA (same length and sequence), was incubated at 37˚C for 10 min. To this mixture, 1 μl of Rad51 (various concentrations) was added, and the solution was incubated for 15 min. The complexes were fixed with 1 μl of 1% glutaraldehyde at 37˚C for 15 min. The products were fractionated through a 1% Seakem GTG agarose (FMC BioProducts) gel in 0.5x TBE buffer for 2 hr at 3.3 V/cm. The gel was dried, exposed to an imaging plate, and visualized using an FLA7000 image analyzer (Fuji Film).

Rad52$^{233\text{-}504}$ DNA binding assay

An 8-μl reaction mixture, containing 1 μl of 10 mM DTT, 1 μl of 10 μM ^{32}P-labeled 60-mer ssDNA (5' GGA ATT CGG TAT TCC CAG GCG GTC TCC CAT CCA AGT ACT AAC CGA GCC CTA TGC TGC TTG 3') or dsDNA (same length and sequence), was preincubated at 37˚C for 10 min. A 2-μl aliquot of Rad52$^{233\text{-}504}$ (various concentrations) was added, and the reaction was incubated for 15 min. The products were fractionated through a 1% Seakem GTG agarose (FMC BioProducts) gel in 0.5x TBE buffer for 2 hr at 3.3 V/cm. The gel was dried, exposed to an imaging plate, and visualized using an FLA7000 image analyzer (Fuji Film).

Addition of Rad52$^{233\text{-}504}$ to preformed Rad51-DNA complexes

A 7-μl reaction mixture, containing 2 μl of 10x reaction buffer (150 mM Mops-KOH, pH 7.3, 100 mM magnesium acetate, 100 mM NaCl, 5 mM DTT), 1 μl of 20 mM ATP, and 1 μl of 10 μM Rad51, was incubated at 37˚C for 10 min. To this mixture, 1 μl of 10 μM ^{32}P-labeled 60-mer ssDNA (5' GGA ATT CGG TAT TCC CAG GCG GTC TCC CAT CCA AGT ACT AAC CGA GCC CTA TGC TGC TTG 3') or dsDNA (same length and sequence) was added, and the solution was incubated for 15 min. A 2-μl aliquot of Rad52$^{233\text{-}504}$ (various concentrations) was then added, and the reaction was incubated for 15 min. The complexes were either fixed with 1 μl of 1% glutaraldehyde, or treated with 2 μl of deproteinization solution (0.5% SDS, 10 mg/ml proteinase K) at 37˚C for 15 min. The products were fractionated through a 1% Seakem GTG agarose (FMC BioProducts) gel in 0.5x TBE buffer for 2 hr at 3.3 V/cm. The gel was dried, exposed to an imaging plate, and visualized using an FLA7000 image analyzer (Fuji Film).

CD spectroscopy

CD spectra of Rad52$^{233\text{-}504}$ and its point mutants were recorded at 20˚C, using a Jasco J-820 spectrometer (Jasco). Proteins were prepared as 0.2 mg/ml solutions in 8 mM Hepes-KOH, pH 7.5, 60 mM KCl, 0.2 mM EDTA, 0.8 mM 2-mercaptoethanol, and 4% glycerol.

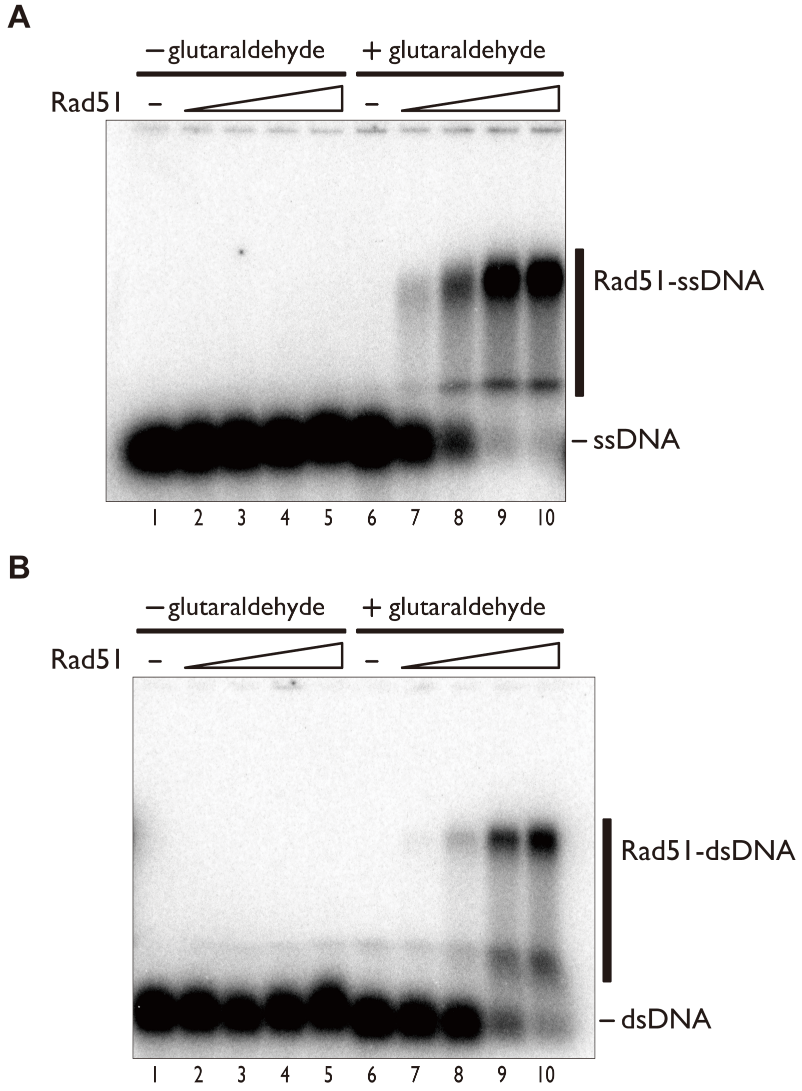

Supplementary Figure S1

Supplementary Figure S1. Visualization of Rad51-DNA complexes by glutaraldehyde crosslinking. Various concentrations of Rad51 were incubated with either 1 μM ssDNA (**A**) or 1 μM dsDNA (**B**). The complexes were either directly fractionated through the agarose gel (lanes 1-5), or fixed with glutaraldehyde, and then fractionated through an agarose gel (lanes 6-10). The Rad51 concentrations were 0.125 μM (lanes 2 and 7), 0.25 μM (lanes 3 and 8), 0.5 μM (lanes 4 and 9), and 1 μM (lanes 5 and 10).

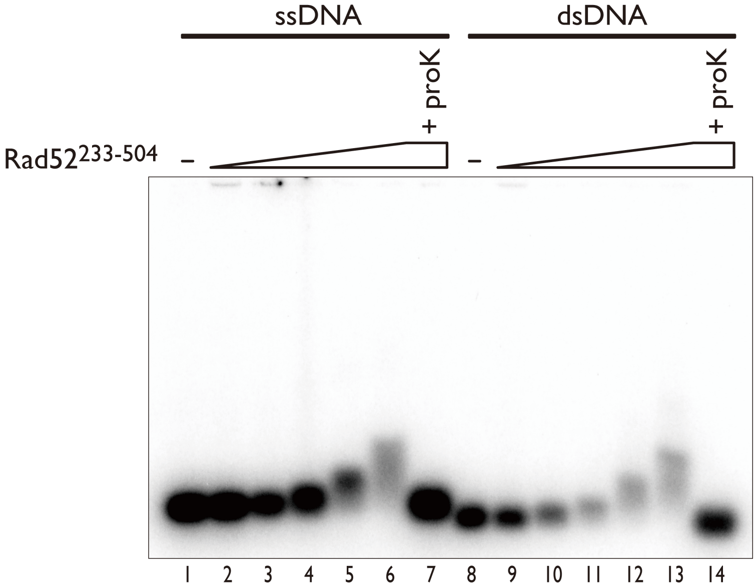

Supplementary Figure S2

Supplementary Figure S2. DNA binding activity of Rad52$^{233\text{-}504}$. Various concentrations of Rad52$^{233\text{-}504}$ were incubated with either 1 μM ssDNA (lanes 2-7) or 1 μM dsDNA (lanes 9-14). The complexes were fractionated through an agarose gel. The Rad52$^{233\text{-}504}$ concentrations were 0.5 μM (lanes 2 and 9), 1 μM (lanes 3 and 10), 2 μM (lanes 4 and 11), 4 μM (lanes 5 and 12), and 8 μM (lanes 6, 7, 13, and 14). To demonstrate that Rad52$^{233\text{-}504}$ is free of nuclease contamination, the Rad52$^{233\text{-}504}$-DNA complexes were treated with proteinase K, and the deproteinized DNA was fractionated through the gel to confirm that there was no loss of signal (lanes 7 and 14).

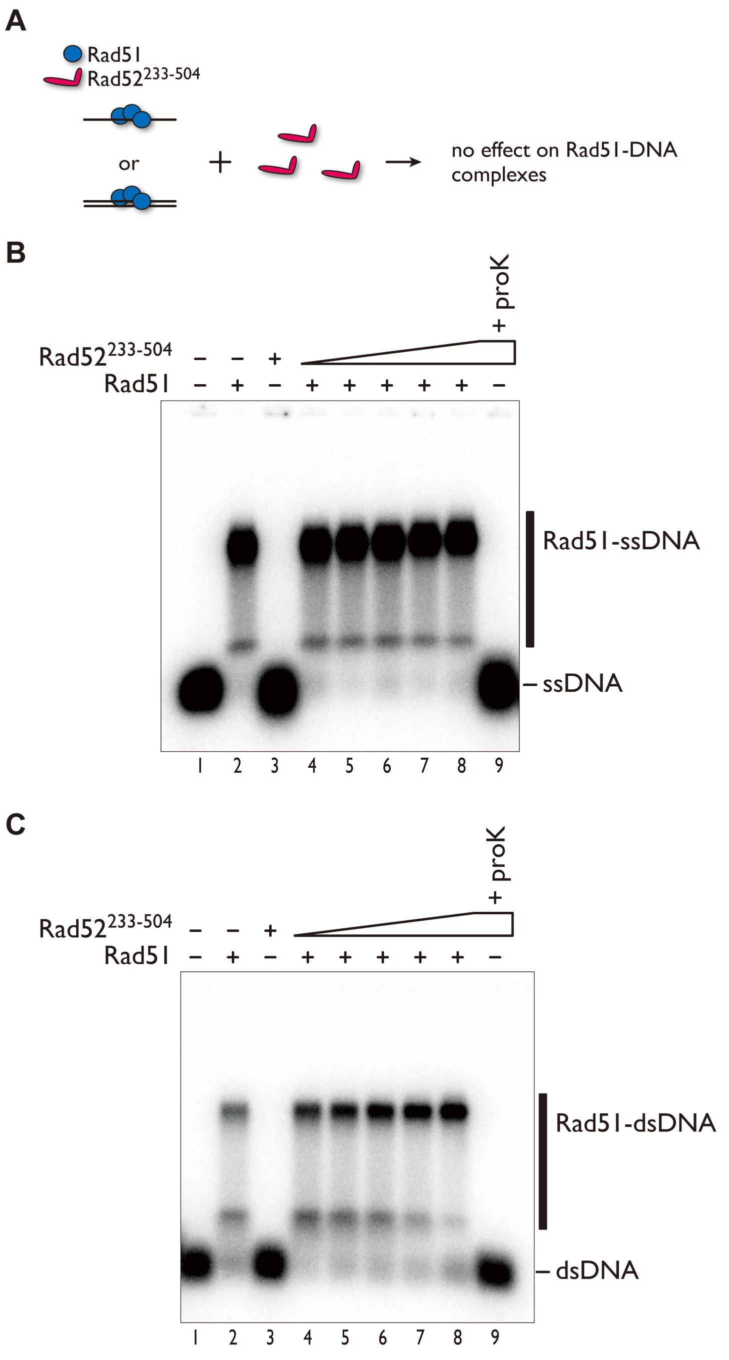

Supplementary Figure S3

Supplementary Figure S3. Effects of Rad52$^{233\text{-}504}$ on preformed Rad51-ssDNA and Rad51-dsDNA complexes. (**A**) Schematic representation of the assay. Various concentrations of Rad52$^{233\text{-}504}$ were added to preformed Rad51-ssDNA (**B**) or Rad51-dsDNA (**C**) complexes (lanes 4-8). The products were stabilized by glutaraldehyde fixation, and fractionated through an agarose gel. The Rad52$^{233\text{-}504}$ concentrations were 0.5 μM (lane 4), 1 μM (lane 5), 2 μM (lane 6), 4 μM (lane 7), and 8 μM (lanes 3, 8, and 9). Lanes 3 and 9 are identical, except for the addition of proteinase K instead of glutaraldehyde.

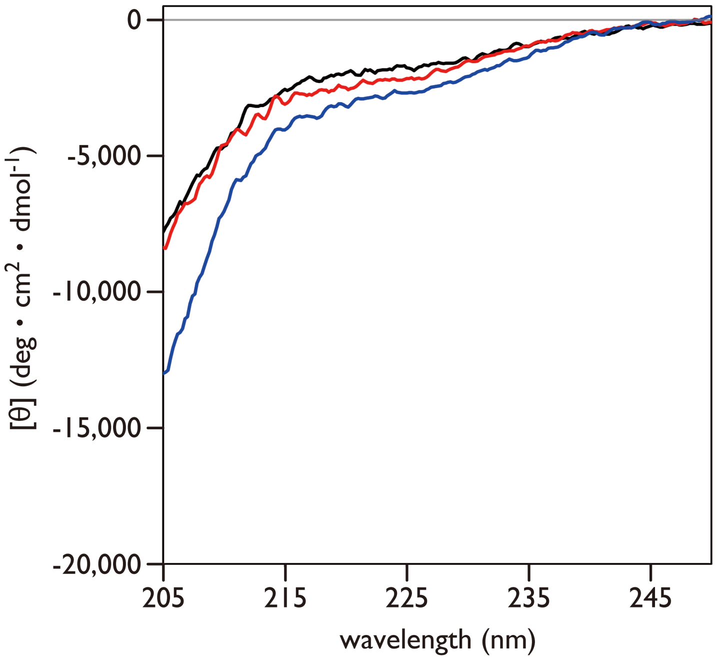

Supplementary Figure S4

Supplementary Figure S4. Circular dichroism spectra of Rad52$^{233\text{-}504}$ and its point mutants (F349A and Y409A). The spectra of Rad52$^{233\text{-}504}$, Rad52$^{233\text{-}504}$ F349A, and Rad52$^{233\text{-}504}$ Y409A are colored black, red, and blue, respectively.
